# Host Evolutionary Lineage Shapes Assembly, Network Topology, and Metabolic Potential of Coral Skeletal Endolithic Microbiomes

**DOI:** 10.3390/microorganisms14010195

**Published:** 2026-01-15

**Authors:** Chuanzhu Bai, Huimin Ju, Jian Zhang, Jie Li

**Affiliations:** 1 State Key Laboratory of Tropical Oceanography, CAS Key Laboratory of Tropical Marine Bio-Resources and Ecology, South China Sea Institute of Oceanology, Chinese Academy of Sciences, Guangzhou 510301, China; 15779226849@shu.edu.cn (C.B.); juhuimin17@mails.ucas.ac.cn (H.J.); zhangjian@scsio.ac.cn (J.Z.); 2 Marine Geological Survey of Jiangsu Province, Jiangsu Geological Bureau, Nanjing 210007, China; 3 Xisha Marine Environmental National Observation and Research Station, Sansha 573199, China; 4 Sanya National Marine Ecosystem Research Station, Tropical Marine Biological Research Station in Hainan, Chinese Academy of Sciences, Sanya 572000, China

**Keywords:** coral skeleton, host phylogeny, endoliths, co-occurrence network, nitrogen cycling, skeletal morphology

## Abstract

Evolutionary history of the host may influence the skeletal morphology of scleractinian corals. However, its effects on the assembly and function of endolithic microbiomes remain unknown. We analyzed bacterial and archaeal microbiomes from the coral skeleton by using 16S rRNA gene sequencing. We collected the samples of seven coral genera distributed among the diverse “Complex” and “Robust” clades. In this study, bacterial α-diversity was significantly higher in the Complex clade relative to the Robust clade. Archaea, on the other hand, remained stable and showed no significant differences between the two host clades, and were most abundantly Nanoarchaeota and Thermoproteota. Analysis of the network topologies showed that network structures were different between the Complex group and the Robust clade. The Robust clade formed a dense and closely knit network among bacteria and archaea. The Com-plex group formed a more modular network structure. Functional predictions further highlighted lineage-specific metabolic strategies. Enrichment was apparent in both nitrification genes (*amoB*, *amoC*) and denitrification genes (*nirK*, *nirS*) in the Complex clade. This suggests that the coupling of these nitrogen cycles is possible. The opposite was observed for the Robust clade, which had low potential for both types of nitrogen cycling. This reflects the degree of diffusion limitation in the more massive skeleton of this host lineage. Overall, species evolutionary lineage is a pre-eminent driver for the selective filtering of endolithic assembly. It generates discrete skeletal micro-niches on which microbial strategies diverge. In particular, Complex corals favor fast metabolic flux, and Robust corals favor strong network connectivity.

## 1. Introduction

Tropical reef ecosystems owe much to the ecosystem engineers—scleractinian corals [1,2]. However, scleractinian corals are not autonomous beings but “holobionts” composed of a cnidarian host, Symbiodiniaceae, and a microbiota, represented by a complex consortium of symbiotic bacteria, archaea, fungi, and viruses [3]. This diverse microbial community is key in determining the well-being of the holobiont, supplying it with essential processes (nutrient cycling, pathogen protection, thermal tolerance, among others) [4,5,6]. In the past, the microbiome in corals has been studied primarily in the surface mucus layer and in host tissues [7,8]. These two compartments are easily accessible and are also highly biologically active. But the coral calcium carbonate skeleton represents the greatest total mass in the coral [9]. However, despite its substantial volume, this component is not commonly studied in microbial surveys. Indeed, it is frequently described as an inert physical support used by the coral rather than a part of the host biological compartment [10]. Further observations indicate that the microbiome inhabiting skeletons (endoliths) could be as such separate from those living tissue. This area of coral skeletons’ microbiome can not be ignored if we claim a holistic understanding of the functioning of coral [11].

The coral skeleton represents a specialized and intricate micro-habitat, functionally equivalent to the “plastisphere” in polluted environments or the “rhizosphere” in soils [10,11,12,13]. While the mucus or tissue is ever-changing and fluid, the skeletal matrix represents a solid, protected, physical niche [10,11], colonized by microbial guilds, the so-called endoliths, including “euendoliths”, the actively boring microorganisms of the carbonate and “chasmoendoliths”, which reside in already existing pores and crevices [14,15]. The inside of the skeleton is not inert. It is instead likely an environment of steep physicochemical gradients [11,16]. Attenuation of light is very rapid from the skeletal surface to the inside of the skeleton and can lead to a vertical stratification for phototrophs such as *Ostreobium* [17,18]. Importantly, if water exchange within skeletal pores is limited, then the oxygen can become depleted locally [19]. Microsensor experiments have demonstrated that within skeletal cavities, the conditions can be anoxic or hypoxic during the hours of darkness when respiration is elevated beyond photosynthesis [19,20]. These variable redox conditions set up contrasting metabolic zones, and therefore, the skeleton can be a microenvironment for transformations of biogeochemical importance. Nitrification, denitrification, and anaerobic ammonium oxidation (anammox) have been recorded within the skeleton [21,22,23]. These metabolisms matter, possibly controlling the N budget of the holobiont and the water chemistry of the calcifying fluid [24,25]. Therefore, the physical structure of the skeleton constrains the distribution of microbial metabolic niches.

Coral skeletal morphology is not random. It is deeply shaped by the host’s evolutionary history [26]. Phylogenetic analyses using mt genes divide scleractinian corals into two divergent lineages, namely, the “Complex” clade and “Robust” clade [27]. These two clades split approximately 250 million years ago, and these two clades follow different biomineralization strategies [28]. The “Complex” clade usually adopts an expedited growth and porous construction strategy. Their skeletons are typically lightly built with often complex branching, reticulate morphologies [29,30]. The structure in general has a high surface to volume ratio, theoretically allowing for porewater flux [31]. By contrast, the “Robust” clade builds heavily calcified skeletons that can be quite massive [32]. Though bulk porosity metrics may be different, the micro-structure of Robust corals, however, comprises thick skeletal walls and extremely detailed corallite morphology [33]. This morphological phenotype may enact distinct sets of physical constraints on internal diffusion and fluid flux [34]. Yet, one of the crucial knowledge holes remaining is that we do not yet understand how these evolutionarily selected skeletal phenotypes influence the assembly of endolithic communities [7]. For example, does skeletal morphology constitute a sorting mechanism for certain microbial taxa? Moreover, what could different skeletal permeabilities (e.g., how diffusion limitations in massive colonies constrain microbial networks)? The relevance of host phylogeny, skeletal structure, and endolithic function in driving interactions has been overlooked in existing research [35]. This relevance is critical for comprehension of how coral lineages respond to environmental disturbance through their microbiomes.

To fill these gaps, here, we have comprehensively explored endolithic microbiomes of two of the primary coral evolutionary lineages, sampled seven representative genera (four Complex, three Robust) at Luhuitou Reef, a site characterized by high coral diversity and well-documented skeletal density data. This site provides an ideal natural laboratory to test how divergent skeletal phenotypes (Complex vs. Robust) influence endolithic assembly. With integration of 16S rRNA gene amplicon sequencing, co-occurrence network analysis, and functional prediction (PICRUSt2). The specific aims of this study were to address (1) whether the evolutionary history of the host influences substantial divergence of endolithic bacterial and archaeal diversity; and (2) whether skeletal constraints (i.e., modularity vs. connectivity) modulate the topology of the microbial interaction networks and the potential biogeochemistry (in particular, nitrogen and sulphur cycling) within those networks. A priori, we expected the clearly divergent skeletal approaches of Complex and Robust corals to promote divergent micro-niches. We suggest that porous, permeable, skeletal forms (Complex) facilitate high metabolic exchange and modular network selection, while diffusion-limited skeletons (Robust) select for high network connectivity and cooperative means to survival.

## 2. Materials and Methods

### 2.1. Study Sites and Sample Collection

Fieldwork was undertaken from March to April 2022 at Luhuitou Reef of Sanya Bay in Hainan, China (18°12′ N, 109°28′ E). Scleractinian coral samples were collected by SCUBA diving. The water depth ranged from 3 to 5 m during the period of fieldwork. During the sampling period, the water temperature, dissolved oxygen saturation, and pH during the sampling period were 26.6–27.1 °C, 99.6%, and 8.16, respectively. Seven coral species covering seven genera were chosen as sampling subjects in this study (Figure S1) [36]. Identification was primarily conducted at the genus level to ensure taxonomic accuracy, as species-level resolution in some scleractinian groups is limited by morphological plasticity and incomplete coverage in marker gene database. To achieve statistical robustness, we collected a minimum of three replicate colonies from each genus. To avoid clonality, different colonies were collected. All sampling was performed quickly to avoid causing physiological stress to the corals. Collected coral fragments were washed with sterile seawater to dislodge most loosely attached microbes, photographed, identified, and each wrapped individually in sterile aluminum foil. All samples were instantly snap-frozen in liquid nitrogen and stored at −80 °C freezer for long-term storage.

### 2.2. DNA Extraction and Sequencing

Coral samples were broken to a consistent size as determined by their morphology (branching: 3 cm × 3 cm × 4 cm, massive: 3 cm × 3 cm × 4 cm, and laminar: 6 cm × 6 cm). Coral tissue from the skeleton was removed through high pressure air-water spray system (adapted from a dental irrigator) in a sterile containment bag [37]. The skeletons were washed in 0.22 μm filtered sterile seawater (FSW) to thoroughly remove remaining tissue. Cleaned skeletons were transferred to fresh sterile bags, ground to a slurry with a sterilized hammer, and moved into 15 mL centrifuge tubes. The fragments of the skeleton were washed with FSW three times vigorously (20 min each) to decant away residual surface material. The resulting skeleton homogenate was centrifuged at 12,000× *g*, 10 min, 4 °C, and the pellet was retained for DNA extraction. Genomic DNA was isolated from skeletal pellets by using the DNeasy PowerSoil Pro Kit (QIAGEN, Germantown, MD, USA) according to the manufacturer’s procedure. Bead beating was carried out immediately before DNA extraction at 4 °C for 10 min to break the sample’s cells properly. A blank was run throughout the extraction procedure to rule out contamination. The quality of DNA were measured in a NanoDrop 2000 spectrophotometer (Thermo Fisher Scientific, Waltham, MA, USA), and the precise concentration was determined using a Qubit™ 4.0 Fluorometer (Thermo Fisher Scientific, USA). The integrity of the isolated DNA was confirmed through 0.8% agarose gel electrophoresis. High-purity DNA (A260/280: 1.8–2.0) was kept at −80 °C.

For the bacterial community, full length 16S rRNA gene was amplified by universal primers 27F (5′-AGRGTTYGATYMTGGCTCAG-3′) and 1492R (5′-RGYTACCTTGTTACGACTT-3′) [38]. A pre-experiment was performed to select the optimal PCR cycle number to guarantee sufficient yield of the PCR product with minimum amplification bias. This slightly higher cycle number was necessitated by the low microbial biomass of the skeletal samples and the potential presence of inhibitory substances common in benthic matrices, to ensure sufficient amplicon yield for downstream sequencing. The total reaction volume was 20 μL, consisting of 0.8 μL DNA template (10 ng), 10 μL 2× Pro Taq Master Mix, and 0.8 μL of each primer (5 μM). The thermocycle program included initial melting (95 °C for 3 min); 29 cycles (95 °C for 30 s, 53 °C for 30 s, 72 °C for 45 s) and final extension (72 °C for 10 min). Primer sequences were tagged with specific barcodes to multiplex the samples. The duplicate PCR products were combined, and both nucleic acids and PCR products were visualized on 2% (*w*/*v*) agarose gel, subsequently purified with AxyPrep DNA Gel Extraction Kit (AXYGEN, Union City, CA, USA), and assessed using the QuantiFluor™-ST (Promega, Madison, WI, USA) for quantification. PacBio Sequel II sequencer platform (Pacific Biosciences, Menlo Park, CA, USA) used to sequence SMRTbell libraries; circular consensus sequencing (CCS) reads were generated. For the archaeal community, amplification of the V4 region of the 16S rRNA gene was performed using primers Arch519F (5′-CAGCCGCCGCGGTAA-3′) and Arch915R (5′-GTGCTCCCCCGCCAATTCCT-3′) [39], with the same reaction composition and thermal cycling parameters as for bacteria (29 cycles). The selection of bacterial and archaeal primers was to leverage the high resolution of full-length 16S sequencing for bacteria while ensuring robust and comparable archaeological coverage using widely validated marine archaea V4 primers. Libraries were sequenced by using NEBNext^®^ Ultra™ II DNA Library Prep Kit (New England Biolabs, Ipswich, MA, USA) on the Illumina NovaSeq 6000 platform (Illumina, Inc., San Diego, CA, USA) with a paired-end 250 bp sequencing strategy.

### 2.3. Bioinformatics

Raw subreads were converted to CCS reads using PacBio SMRT Link (v8.0), and subsequent analysis was performed with the QIIME 2 pipeline (v2022.11) [40]. Trimmomatic (v0.33) [41] was used for quality filtering, while primer sequences were removed with Cutadapt (v3.4) [42]. DADA2 (v1.26) QIIME 2 plugin was used for denoising, chimera removal, and generation of Amplicon Sequence Variants (ASVs). In case of archaea (Illumina data), we quality-filtered the paired-end reads with fastp and trimmed the primers with Cutadapt. Using the DADA2 plugin, we merged the paired-end reads and inferred ASVs. We assigned taxonomic annotation to ASVs with a Naive Bayes classifier trained on the SILVA 138 SSURef NR99 database [43]. In order to maintain data quality, sequences annotated as mitochondria, chloroplasts, and non-target domains were excluded. We also removed any potential contaminants observed in negative controls, and lastly, to account for sampling effort, we rarefied the ASV tables to the lowest sequencing depth (9480 reads for bacteria and 2913 reads for archaea) across samples before downstream analysis of diversity.

### 2.4. Statistical Analysis

All data analyses and visualization were conducted within the R environment (v4.3.0). The phylogenetic tree of hosts was built and visualized by the R packages ggtree (3.10.1), treeio (1.26.0), and ape (5.8-1), according to mitochondrial sequences (COI) [27,32] and then rooted with *Nematostella vectensis*. For the microbial community, we obtained Alpha diversity indices (Chao1 and Shannon), calculated with the vegan (2.7-1) package. Beta diversity was calculated according to Bray–Curtis dissimilarities. PCoA was applied to visualize compositional differences in community members, with results regarding the effect of host lineages tested by PERMANOVA with 9999 permutations using the function adonis2 from the vegan package. The Wilcoxon rank-sum test with the package ggpubr (0.6.1) was implemented to compare the differences in Alpha diversity between the complex and robust clades. Alluvial plots to illustrate taxonomic composition were performed with the ggalluvial R package. To determine if a specific microbial taxon corresponds to one host clade, Linear Discriminant Analysis Effect Size (LEfSe) was performed (LDA score > 2.0, *p* < 0.05). Correlation of skeletal porosity with microbiome richness was performed with Pearson correlation analysis and visualized with linear regression models.

We generated cross-domain co-occurrence networks using the igraph (2.1.4) and psych (2.5.6) packages. To provide a conservative estimate, only ASVs (mean relative abundance > 0.1% and prevalence > 30%) were retained; pairwise Spearman’s rank correlations were used, and edges were retained if the correlation coefficient |r| > 0.6 and FDR-adjusted *p*-value < 0.05. Network topological properties (e.g., connectedness, assortativity, and transitivity) are provided, including parameters that can be identified, such as transitivity and entropy, and for other properties that are not directly detected, including modularity and centrality. Network parameters allow us to conclude the nature of relationships between bacteria at the ASV level (such as a connection, lack of association, co-occurrence, or shared habitat requirements). For every host clade, modularity, graph density, and average path length were determined. Within- and among-module connectivity (Zi and Pi, respectively) determined the topological roles of individual nodes in the networks, which were ranked in peripheral, connector, module hub, and network hub classes [44,45]. All networks were plotted with Gephi (v0.10.1).

PICRUSt2 (v2.5.2) predicts functional potential of bacterial/archaeal communities based on 16S rRNA gene sequences. The predicted counts of KEGG Orthology (KO)s were normalized to copies per million (CPM) [46]. Functional genes that are relevant to each of the main sources of metabolism for nitrogen and sulfur cycling are extracted and aggregated into metabolic pathways. Host clade between predicted functions is tested for statistically significant differences with the Wilcoxon rank-sum test. The predicted functional profiles are plotted with bubble and boxplots using the ggplot2 (4.0.1) package.

## 3. Results

### 3.1. Phylogenetic Context and Sequencing Data

**Figure 1:**
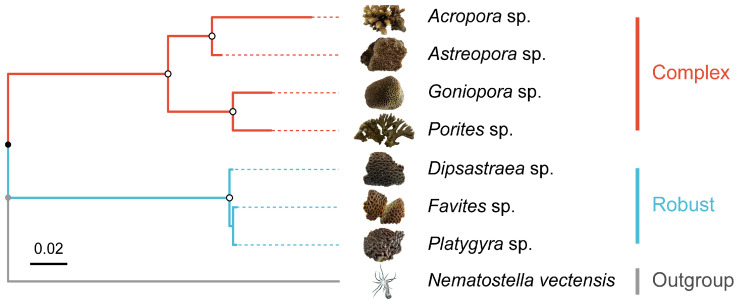
Host phylogenetic framework. Maximum Likelihood phylogenetic tree of the seven sampled coral genera based on mitochondrial COI sequences. Branches are colored by evolutionary clade: Complex (red) and Robust (blue). *Nematostella vectensis* was used as the outgroup. The dotted lines indicate the correspondence between coral images and the phylogenetic branches.

We analyzed the endolithic microbial communities of seven scleractinian coral genera collected from Luhuitou Reef (sample details and GenBank accession numbers are listed in Table S1). Rarefaction curves for both bacterial and archaeal communities reached saturation, indicating that the sequencing depth was sufficient to capture the majority of microbial diversity (Figure S2). Based on mitochondrial COI gene sequences, the phylogenetic tree clustered the seven coral genera into two distinct evolutionary lineages: the complex clade (*Acropora*, *Astreopora*, *Goniopora*, *Porites*) and the robust clade (*Dipsastraea*, *Favites*, *Platygyra*) (Figure 1).

### 3.2. Host Lineage Drives Distinct Diversity Patterns

**Figure 2:**
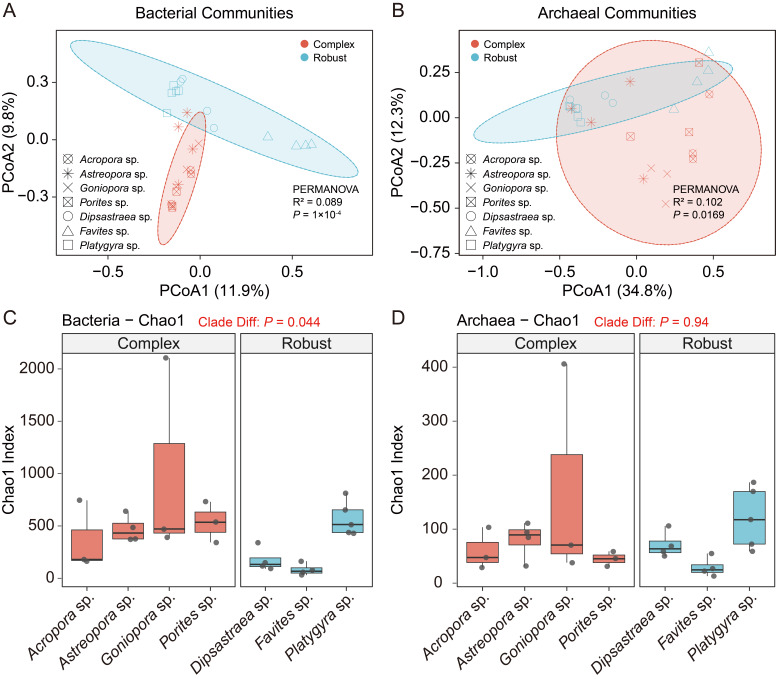
Patterns of diversity of endolithic bacterial and archaeal communities along host clades. (**A**,**B**) PCoA of the dissimilarities using the Bray–Curtis distance of bacterial (**A**) and archaeal communities (**B**). The 95% confidence interval is shown by ellipses. R^2^ and *p*-values were tested with PERMANOVA. (**C**,**D**) Boxplots of richness indices of Chao1 for bacterial (**C**) and archaeal (**D**) communities. Comparison between clades was made by the Wilcoxon rank-sum test. Observe that there is a large variance among bacteria but little variance among archaea.

PCoA of the Bray–Curtis distances indicated that there was a clear separation in community structure between the two host clades for bacteria (PERMANOVA, R^2^ = 0.089, *p* < 0.001) and archaea (PERMANOVA, R^2^ = 0.102, *p* = 0.017) (Figure 2A,B). There was also a significant difference in α-diversity between the two clades (Table S2). In particular, the bacterial Chao1 richness index in the complex clade was greater than in the robust one (Figure 2C, *p* = 0.044), although the Shannon diversity indices did not change between the two clades for the bacteria (*p* = 0.14) and archaea (*p* = 0.26) (Figure S3, Table S2). Likewise, no significant difference was found in the Chao1 index for the archaeal communities between the two clades (Figure 2D, *p* = 0.94). Bacterial richness at the genus level ranged widely within the complex clade (high *Goniopora* values, Figure 2C) and was generally low among all genera for archaeal richness (Figure 2D). We aimed to better assess the possible physical drivers here, too, by analysing skeletal porosity estimated from density values (Table S3). We find no significant linear correlation (on the level of the individual samples) between alpha diversity and porosity (Figure S4). Additionally, Mantel tests revealed that skeletal porosity was significantly correlated with bacterial community structure (*p* < 0.05), whereas no significant correlation was found for archaeal communities (*p* > 0.05) (Table S4). However, these correlations indicate that skeletal porosity alone is not the sole determinant of the observed microbial patterns, and the statistical power is limited by the inherent spatial sensitivity of these measures.

### 3.3. Taxonomic Composition and Biomarkers

**Figure 3:**
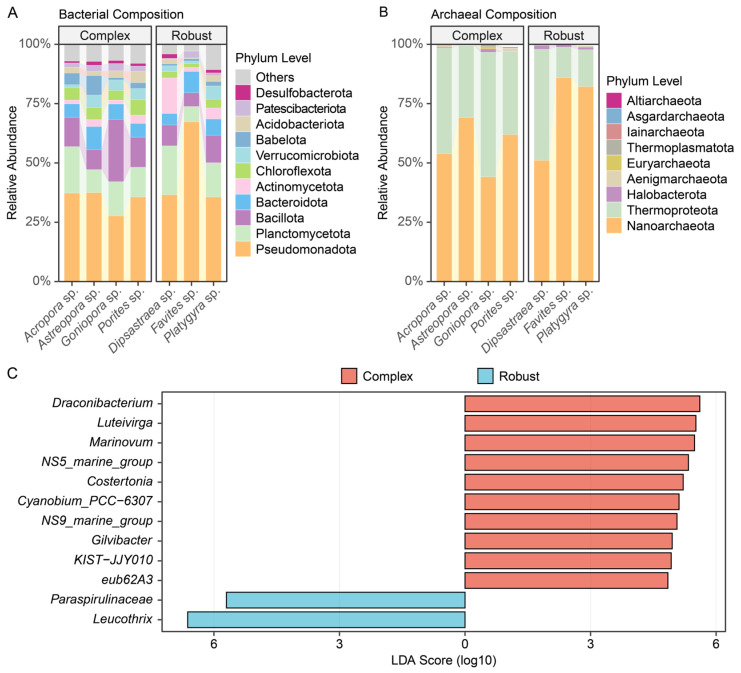
Taxonomic composition and differential abundance analysis. Alluvial plots showing the relative abundance of dominant bacterial (**A**) and archaeal (**B**) phyla across individual coral genera. (**C**) LEfSe analysis identifying bacterial genera significantly enriched in complex (red) or robust (blue) clades (LDA score > 4.0, Top 10 shown). No archaeal genera were significantly differentially abundant (LDA < 2.0).

The taxonomic composition of endolithic communities displayed clear patterns between host lineages (Figure 3 and Figure S5). At the phylum level, Pseudomonadota, Planctomycetota, and Bacillota were the most abundant in the case of bacteria, although relative abundances differ between complex and robust clades (Figure 3A, Table S5). In particular, the Pseudomonadota mean relative abundance was higher in the robust (45.7%) than in the complex (34.8%) clade. Other major phyla, including Planctomycetota, Bacillota, and Bacteroidota, collectively accounted for 35.2% of the community in the Complex clade and 29.7% in the Robust clade. Chloroflexota and Babelota exhibited an increased proportion in the Complex clade (5.2% and 4.4%, respectively) compared to the Robust clade (2.7% and 1.3%, respectively) (Table S5). On the archaea side, members of both clades were predominantly represented by Nanoarchaeota and Thermoproteota (Figure 3B). In contrast to bacteria, the archaeal relative abundances appeared quite constant across both clades and genera (Figure 3B and Figure S5, Table S6). LEfSe allowed us to detect specific bacterial genera mostly enriched in each clade (LDA score > 4.0, Figure 3C). Genus *Draconibacterium*, *Luteivirga*, and *Marinovum* were enriched in the complex clade, and *Paraspirulinaceae* and *Leucothrix* were enriched in the robust clade (Figure 3C). No differential abundance of archaeal genus was found between complex and robust clades, reflecting a relatively uniform archaeal distribution across host clades.

### 3.4. Topological Differences in Cross-Domain Networks

**Figure 4:**
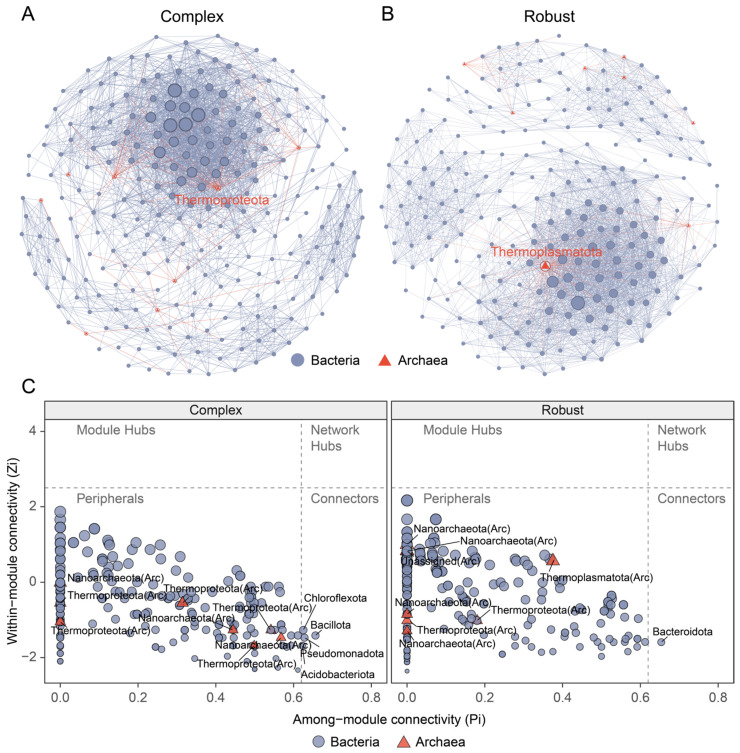
Cross-domain co-occurrence networks and topological roles of endolithic communities. (**A**,**B**) Network graphs for complex (**A**) and robust (**B**) clades constructed from ASV correlations (Spearman |r| > 0.6, *p* < 0.05). Nodes represent ASVs, sized by degree. Edges represent significant correlations. (**C**) Zi-Pi plots classifying node roles based on within-module (Zi) and among-module (Pi) connectivity.

We constructed two cross-domain co-occurrence networks at the ASV level for complex and robust clades (Figure 4A,B). Visualizations colored by taxonomy and modularity highlighted the distinct community structures within each network (Figure S6). In both networks, the relationships among microorganisms were predominantly characterized by positive associations (>99% positive edges) (Table S7). The network in the robust clade contained 3127 edges, significantly more than that in the complex clade (2649 edges), despite a similar number of nodes (230 vs. 248) (Table S7). Compared with the complex network, the robust network exhibited a higher average degree (27.19 vs. 21.36), graph density (0.119 vs. 0.086), and clustering coefficient (0.641 vs. 0.578) (Table S7). In both networks, archaea were represented by a stable set of 8 nodes (Figure 4C, Table S8). No module hubs (Zi > 2.5) were identified in either network. The complex network contained four bacterial module connectors (Chloroflexota, Bacillota, Pseudomonadota, and Acidobacteriota), whereas the robust network contained only one bacterial module connector (Bacteroidota) (Figure 4C, Table S8).

### 3.5. Potential Metabolic Functions

**Figure 5:**
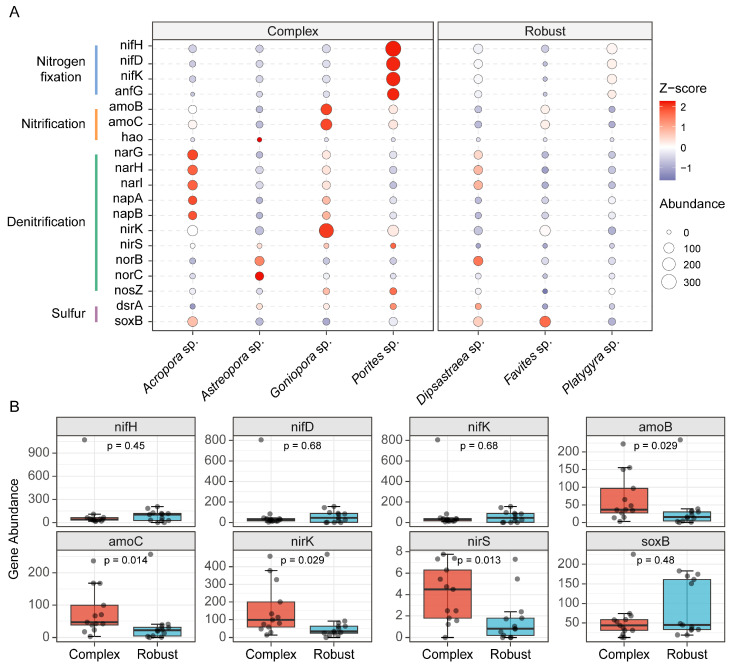
Predicted functional and differential nitrogen metabolism potential between coral host lineages. (**A**) Bubble plot profiling the relative abundance and enrichment patterns of key nitrogen and sulfur functional genes across seven coral genera. Genes are grouped by metabolic pathways. Circle size indicates the mean abundance (CPM, Copies Per Million), and color intensity represents the Z-score of relative intensity. (**B**) Boxplots comparing the abundance of key metabolic marker genes between Complex (red) and Robust (blue) clades. Statistical significance was determined using the Wilcoxon rank-sum test (*p* values shown). The box represents the interquartile range (IQR), the center line denotes the median, and whiskers extend to 1.5 × IQR.

Given the differences seen in the structure of the microbial communities associated with the respective host lineages, we used PICRUSt2 to predict the functional potentials of the different skeletal microbiomes, focusing on bacterial carbon and nitrogen, and sulphur cycling (Figure 5, Table S9). Genus-level profiling revealed that diazotrophic potential was unevenly distributed and host genus-driven. Figure 5A demonstrates the functional landscape, for example, high *nifH* and *nifD* abundances in the genus *Porites* and *Acropora* (Complex clade), while other genera showed comparatively less potential. In terms of sulfur metabolism, no significant overall statistical difference was found between the Complex and Robust clades for sulfur-oxidizing (*soxB*, *p* = 0.48) or sulfate-reducing (*dsrA*) genes (Figure 5B); however, we did find host-specific signatures. This lack of clade-level divergence in sulfur cycling and nitrogen fixation potential suggests that while nitrogen turnover is highly lineage-dependent, these specific pathways may represent conserved functional core components of the skeletal microbiome.

We found evidence for marked lineage-specific variation in nitrogen flux potential. The potential for nitrogen cycling was substantially greater in the Complex than the Robust clade (Figure 5B). Particularly, we observed significant enrichment for some key genes of nitrification in the Complex skeleton, such as *amoB* (*p* = 0.029) and *amoC* (*p* = 0.014). Similarly, denitrification potential was also significantly increased in Complex lineage, such as abundance of genes that code for nitrite reductase, *nirK* (*p* = 0.029) and *nirS* (*p* = 0.013) (Figure 5B). Unlike the clade-wide conservation of nitrification and denitrification potentials, *nifH*-mediated nitrogen-fixation potential exhibited no large difference between the two evolutionary lineages (*p* = 0.45; Figure 5B).

## 4. Discussion

### 4.1. Niche Differentiation Driven by Skeletal Morphology

We show here that the α-diversity (Chao1 and Shannon indices) of the bacterial assemblage in the Complex clade was significantly greater than in the Robust clade (Figure 2). This observation implies that the host clade imposes an important selection pressure upon endolithic community assembly. We postulate that the observed diversity gradient may be associated with the different skeletal architecture resulting from host biomineralization tactics.

Branching complex corals (e.g., *Acropora*) are generally characterized by branching morphologies with a high surface-to-volume ratio [47]. Despite the density of the skeletal material, the branching geometry provides a short diffusion path length. This geometry assists the inflow of seawater and oxygen inside the skeletal matrix [31]. Thus, it generates heterogeneous microenvironments (e.g., gradient of light and oxygen). These heterogeneous micro-niches provide potential coexistence of more varied bacterial taxa, allowing for the support of the “High Metabolic Turnover” strategy in our functional analysis, as per niche theory, with its higher environmental heterogeneity promoting high species richness [48], whereas the Robust clade (e.g., *Favites*) consists of massive growth forms with robust skeletal walls. Although Robust corals have a high bulk porosity, their internal pore structure has high tortuosity (“Maze Effect”) [32,33]. The physical pore structure strongly limits porewater exchange, both chemically through permeability [11,49,50] and biologically through restricted permeability of the Robust skeletons [51], and serves as a powerful environmental filtering mechanism. The flow restrictor lowers the supply of nutrients and establishes a stalling micro-environment, with only specifically adapted taxa being able to persist there. This, presumably robust, filtering environment may influence the reduced bacterial diversity of the Robust clade. Since significant differences were observed in Chao1 richness but not in Shannon diversity, it suggests that host lineage may primarily modulates the rare bacterial taxa rather than the dominant community members. In addition, LEfSe also demonstrated specific biomarkers for each lineage. The Complex clade was enriched for Chloroflexota, which is a taxon often found in marine sediments with a broad variety of heterotrophic lifestyles [52]. In contrast, the Robust clade enriched particular Pseudomonadota. This taxonomic change validates that the morphology of the skeleton not only selects for diversity but also selects for particular phylogenetic groups. These findings illustrate that the coral skeleton is not merely a passive substrate. Rather, the “skeletal plastisphere” that hosts a specific feature actively modulates the “skeletal morphotype” through micro-environmental stability and its resources.

### 4.2. The Conservatism of Endolithic Archaea

In contrast to the lineage-dependent signatures seen in bacteria, archaeal endolith communities were remarkably conservative, showing no major differences in either α-diversity or composition between the Complex and Robust clades (Figure 2D and Figure 3B). This phenomenon indicates that the assembly of archaea is largely decoupled from the host’s specific skeletal form.

Thermoproteota and Nanoarchaeota were the most prevalent archaeal phyla in all samples. Such co-occurrence of these two phyla is not a product of physical filtering but a biological association of these organisms. Thermoproteota were always an abundant population in ocean waters [53], and many species have *amo* gene associated with ammonia oxidation, which is crucial for the oxidation of ammonia during the first step of the nitrification process [54]. Nanoarchaeota, on the other hand, are obligate symbionts/parasites that associate physically with Thermoproteota hosts and draw lipids and amino acids from these hosts [55,56]. Our samples, in turn, show synchronized presence of these archaea, possibly as a result of the tight biological dependency. Why are these archaea unresponsive to skeletal variation? This conservation might arise from methodological constraints, such as the limited taxonomic resolution of the V4 primer set [57], or shared environmental stressors across skeletal types. Furthermore, the possibility of archaeal dormancy or low metabolic activity within the dense carbonate matrix cannot be ruled out, which would result in a stable but less active community signature [58]. Irrespective of whether skeletons are porous (Complex) or massive (Robust), all corals excrete ammonium as metabolic waste [21]. Therefore, there should be a steady and relatively continuous substrate supply for ammonium-oxidizing archaea present in the skeleton. Thus, the archaeal community most likely stands for a “core microbiome” established early in the life-history of a coral [35]. This conclusion is corroborated by network analyses, as archaeal nodes all belonged exclusively to the class of “peripherals” in both networks (Figure 4). They were neither module hubs nor connectors. This topological role suggests that archaea are simply phylum-stable residents that do not orchestrate the wider community structure; they presumably execute specific, conserved metabolic duties (e.g., ammonia oxidation) without much input into the variable bacterial interactions controlled by skeletal micro-niches.

### 4.3. Network Topology Reflects Micro-Environmental Stability

It was apparent from network analysis that the two host lineages demonstrated a pronounced difference in topological patterns, with the physical structure of the Robust skeletons producing a more complex network (demonstrated through higher overall complexity, graph density, and average degree compared to the Complex) (Table S7). We speculate that this increased connectivity is a direct consequence of the physical parameters of the skeleton imposed on the bacterial populations. As detailed in Section 4.1, high tortuosity and diffusion barriers in Robust skeletons give rise to a resource-limited micro-environment [34].

Evolutionary theory, more specifically the Stress-Gradient Hypothesis, states that the biotic interactions change from competing to facilitating at stressful conditions [59]. At the no-flow center of Robust skeletons, the substrate resources (e.g., dissolved organic carbon, ammonium) may become limited due to diffusional limitation [60]. In case of oligotrophic surroundings, cooperative microbial interactions such as syntrophy, cross feeding, have to be applied for survival [61,62,63]. For instance, the metabolic waste of one species is used as a substrate by another. This obligate dependency leads to a “tight-knit” network structure with a high number of connections. This structure is similar to the observation in the “microplastisphere” of inland wetlands, where the unique physical surfaces selected for highly connected microbial consortia [51,64]. The network of the Complex clade has been noted to be defined by lower density and high modularity (Figure 4). This topology represents a community of highly differentiated functional units [45]. The steep but stable Complex skeleton gradients of oxygen and light are available as a function of high permeability [65]. These gradients allow aerobic (e.g., nitrifiers) and anaerobic (e.g., denitrifiers) functional groups to occupy separate spatial niches [21]. By maintaining sufficient resources through porewater advection, the functional groups remain more or less isolated. This potentially allows sparse global communication throughout the community. Such topological dissimilarity has important implications for the stability of a community. Higher modularity, like in the Complex clade, is sometimes an advantage. It can segregate perturbations (say a pathogen invasion, a pH swing) within a module to prevent collapse of the entire ecosystem [66,67,68]. In contrast, the extensively linked structure of the Robust network implies highly efficient resource transfer, yet potential instability. An assault on a single critical taxon may cascade quickly into an entire network, a phenomenon referred to as the “robustness-fragility” trade-off [44,69]. Thus, the “maze-like” skeleton of Robust corals directs the assembly of a mutually beneficial, yet potentially delicate microbial community.

### 4.4. Skeletal Morphology Drives Divergent Metabolic Strategies

**Figure 6:**
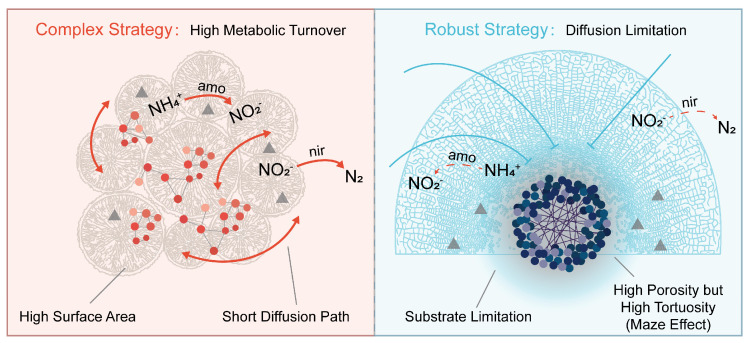
Proposed hypothetical framework for the relationship between coral skeletal morphology and niche partitioning, microbial community assembly, and functional trait expression along host evolutionary lineages. (**Left**) Complex Strategy (High metabolic Turnover): Complex corals (e.g., *Acropora*) exhibit skeletal morphologies with a high surface to volume ratio that induce a short diffusion distance so that seawater and oxygen can diffuse into the skeletal matrix. Thus, the microbiome generates spatially coupled aerobic and anaerobic micro-niches and develops modular, surface-associated networks (scattered red dot clusters). Solid red arrows show coupled cycling of nitrogen (high *amo* and *nir* gene abundances), driving fast cycling of nitrogen to sustain host metabolism. (**Right**) The Robust Strategy (Diffusion Limitation): Robust corals (e.g., *Favites*) have massive growth forms. Even though it is highly porous, this complex inner morphology enforces the “Maze Effect” of high tortuosity. As observed with blocked blue lines, this imposes harsh constraints on the substrate supply (i.e., ammonium supply is hampered). The resulting resource-strained environment hampers maximal nitrogen cycling (red dotted arrows). Nevertheless, it sieves for a unique bacterial community with a well-connected and dense co-occurrence network (central purplish-blue dot clusters) through exhibiting intense biotic interactions to subsist in the sedimentary central stagnant core.

Functional predictions via PICRUSt2 uncovered a clear lineage-based divergence in nitrogen metabolism. The Complex clade demonstrated a significantly amplified potential for nitrogen turnover relative to the Robust clade (Figure 5). Combining these functional insights with our network and morphological data, we propose a hypothetical conceptual model for skeletal niche differentiation (Figure 6).

The skeletal strategy for Complex corals is “High Metabolic Turnover” (Figure 6, Left). Their branching structure forms a large surface-to-volume ratio architecture that reduces the distance for seawater and oxygen diffusion [20]. High porosity prevents permanent zones of stagnation, but allows for aerobic and micro-anaerobic niches to exist concurrently [65]. Such a physicochemical environment allows for parallel enhancement of both nitrifying genes (*amoB*, *amoC*) and denitrifying genes (*nirK*, *nirS*). We propose that Complex skeletons act like “active biofilters”. High metabolic potential for nitrification and denitrification in Complex corals suggests a potential coupling of aerobic and anaerobic nitrogen processes. This coupling might be facilitated by the porous skeletal structure, which creates a mosaic of redox micro-zones. It is likely they drive this coupled nitrification-denitrification, quantified to date for permeable reef sediments [23,70,71], that fast N-cycling could underlie fast skeletal extension rates found in Complex [72]. The Robust clade, on the other hand, follows a “Diffusion Limitation” approach (Figure 6, Right). High local porosity notwithstanding, the giant body size and the convoluted internal structure provide a “maze effect” (high tortuosity) [33], severely limiting the physical ability of external substrates to penetrate inside the coral: the potentiality of nitrogen cycling is thus strongly compromised with respect to Complex corals [34]. Yet this limited resource base explains the assembly of the dense and cooperative microbial communities described in Section 4.3. These communities of lower metabolic throughput might have improved stability against environmental perturbation to explain the “stress-tolerant” life history associated with the giant corals [73]. Notably, we detected no differences between lineages regarding sulfur cycling or nitrogen fixation genes (Figure 5). Rather, these functions were led by particular host genera. This observation implies that even though skeletal physics (lineage-driven) sets the “global” environment (e.g., diffusion rates), particular host-microbe molecular interactions may set specialized metabolic pathways [74].

While our findings add important details to our understanding of the linkage between host evolution and skeletal microbiome, there remain some limitations in our study. First, our functional outcomes were developed on 16S rRNA gene predictions (PICRUSt2). These predictions are, of course, informative but need to be verified in future experiments using metagenomics or meta-transcriptomics to validate the expression of active genes [46]. Second, we targeted our analyses to the skeletal morphology that may drive this phenomenon. The other factors, including the organic matrix composition of their host and the skeleton’s pH, differ between lineages, which can affect the assembly of associated microbiota [11,16,75]. Lastly, the sampling in this study was at a time point. Further studies should thus explore the response of this lineage specific networks to environmental stressors such as ocean warming and ocean acidification. Particularly, whether the “modular” (Complex) or “connected” (Robust) networks are able to hold up better against bleaching events shall be relevant in the predictions about the fate of coral reefs [76].

## 5. Conclusions

Here, our findings suggest that host evolutionary lineage is a significant factor associated with the endolithic microbiome assembly in coral skeletons. By comparing “Complex” and “Robust” clades, we observed that divergent skeletal architectures correspond to distinct microbial ecological strategies. The porous and branching skeletons of the Complex clade appear to support higher metabolic potential and nitrogen cycling, whereas the massive skeletons of the Robust clade possess dense, highly connected microbial networks. While bacteria exhibited lineage-specific patterns, the archaeal community remained remarkably stable across different hosts, likely implying a conserved basal niche. These results point to the coral skeleton as an active “skeletal plastisphere” that modulates micro-environments, rather than a passive support. It should be noted that these relationships are correlative; future research utilizing direct physicochemical sensors and meta-transcriptomics will be essential to further validate the specific metabolic fluxes and causal mechanisms within these skeletal micro-niches.

## Data Availability

The original data presented in the study are openly available in [Figshare] at [https://doi.org/10.6084/m9.figshare.30879713] (accessed on 11 January 2026). The R scripts used for statistical analysis and visualization are available on GitHub (R version 4.3.0) at https://github.com/ChuanZhuBai/Coral-Skeleton-Endoliths-Project (accessed on 11 January 2026).

## Supplementary Materials

The following supporting information can be downloaded at https://www.mdpi.com/article/10.3390/microorganisms14010195/s1. Table S1: Information on coral host species and GenBank accession numbers used for phylogenetic tree construction; Table S2: Alpha diversity indices (Chao1 and Shannon) of endolithic bacterial and archaeal communities for each coral sample; Table S3: Reference values of bulk skeletal density and calculated porosity for the seven scleractinian coral genera; Table S4: Statistical tests linking skeletal porosity with microbial community structure (Beta-diversity); Table S5: Mean relative abundances (±%) of dominant bacterial phyla across different coral genera and evolutionary clades; Table S6: Mean relative abundances (±%) of dominant archaeal phyla across different coral genera and evolutionary clades; Table S7: Topological properties of cross-domain co-occurrence networks constructed from endolithic bacterial and archaeal communities in complex and robust coral clades; Table S8: Topological roles and connectivity scores (Zi and Pi) of microbial nodes identified in the co-occurrence networks of complex and robust coral clades; Table S9. Detailed information of the functional marker genes used for metabolic potential prediction in PICRUSt2 analysis; Figure S1. Morphological features of the seven sampled coral genera; Figure S2: Rarefaction curves of 16S rRNA gene sequences for endolithic bacterial and archaeal communities. Bacterial communities (A); archaeal communities (B); Figure S3: Shannon diversity indices of endolithic microbial communities compared between host evolutionary lineages. Bacterial communities (A); archaeal communities (B); Figure S4: Correlation analysis between coral skeletal porosity and microbial alpha diversity. Bacterial communities (A); archaeal communities (B); Figure S5: Taxonomic composition of endolithic communities at both phylum and family levels. Alluvial plots illustrate the mean relative abundance of dominant (A) bacterial and (B) archaeal phyla, as well as (C) bacterial and (D) archaeal families; Figure S6: Visualization of cross-domain co-occurrence networks colored by taxonomic affiliation and modular structure. Taxonomic affiliation for Complex (A) and Robust (B) clades; modularity structure for Complex (C) and Robust (D) clades. References [31,32,36,77] are cited in the Supplementary Materials.
