# Supplementary material for: Host Evolutionary Lineage Shapes Assembly, Network Topology, and Metabolic Potential of Coral Skeletal Endolithic Microbiomes
Source: Microorganisms. 2026 Jan 15;14(1):195. doi: 10.3390/microorganisms14010195 (PMC12844351; doi:10.3390/microorganisms14010195)
Supplement: Supplementary file 1 [file microorganisms-14-00195-s001.zip › microorganisms-4075643-supplementary.pdf]

Supplementary Material for

Host evolutionary lineage shapes assembly, network topology, and metabolic potential of coral skeletal endolithic microbiomes

Chuanzhu Bai[1], Huimin Ju[1,2], Jian Zhang[1,3], Jie Li[1,4,*]

[1]State Key Laboratory of Tropical Oceanography, CAS Key Laboratory of Tropical Marine Bio-Resources and Ecology, South China Sea Institute of Oceanology, Chinese Academy of Sciences, Guangzhou 510301, China

[2]Marine Geological Survey of Jiangsu Province, Jiangsu Geological Bureau, Nanjing 210007, China

[3]Xisha Marine Environmental National Observation and Research Station, Sansha 573199, China

[4]Sanya National Marine Ecosystem Research Station, Tropical Marine Biological Research Station in Hainan, Chinese Academy of Sciences, Sanya 572000, China

*Corresponding author.

Email address: lijietaren@scsio.ac.cn (J. Li)

This file contains 9 tables and 6 figures.

Table S1. Information on coral host species and GenBank accession numbers used for phylogenetic tree construction.

Clade	Coral genus	Representative Species	Marker Gene	GenBank Accession No.
Complex	*Acropora* sp.	*Acropora tenuis*	COI	MN413778.1
Complex	*Astreopora* sp.	*Astreopora myriophthalma*	COI	MN413848.1
Complex	*Goniopora* sp.	*Goniopora planulata*	COI	MN413757.1
Complex	*Porites* sp.	*Porites lutea*	COI	ON970560.1
Robust	*Dipsastraea* sp.	*Dipsastraea vietnamensis*	COI	ON970511.1
Robust	*Favites* sp.	*Favites paraflexuosa*	COI	EU371694.1
Robust	*Platygyra* sp.	*Platygyra daedalea*	COI	ON970546.1
Outgroup	*Nematostella vectensis*	*Nematostella vectensis*	COI	MH087700.1

Table S2. Alpha diversity indices (Chao1 and Shannon) of endolithic bacterial and archaeal communities for each coral sample.

Index	Complex	Robust	*Acropora* sp.	*Astreopora* sp.	*Goniopora* sp.	*Porites* sp.	*Dipsastraea* sp.	*Favites* sp.	*Platygyra* sp.
Bacteria Reads	21046.15 ± 9985.32	17330.31 ± 4989.99	14513 ± 4778.74	23604.5 ± 12063.01	27186.67 ± 13417.11	18027.67 ± 5456.3	16152.75 ± 8047.78	16681.25 ± 4722.31	18791.6 ± 2218.67
Bacteria Chao1	579.86 ± 492.31	297.92 ± 253.64	361.81 ± 331.69	469.33 ± 126.63	988.83 ± 965.99	536.33 ± 193.68	173.71 ± 112.36	82.31 ± 58.08	569.77 ± 163.39
Bacteria Shannon	5.01 ± 0.96	4.25 ± 1.13	4.2 ± 1.41	4.85 ± 0.41	5.56 ± 1.17	5.47 ± 0.43	3.75 ± 0.56	3.2 ± 0.57	5.48 ± 0.37
Archaea Reads	29261.15 ± 16634.77	40986.08 ± 23862.81	15626.33 ± 12943.4	44887.75 ± 8397.41	21315 ± 16589.7	30006.67 ± 15779.1	48190 ± 16871.64	12434.25 ± 14703.01	58064.4 ± 10049.28
Archaea Chao1	88.47 ± 99.45	77.37 ± 53.74	60.03 ± 38.84	80.23 ± 34.69	171.32 ± 203.38	45.05 ± 14	70.83 ± 24.61	29.3 ± 18.09	121.04 ± 56.81
Archaea Shannon	2.26 ± 1	1.8 ± 0.7	2.44 ± 0.61	1.92 ± 0.55	2.82 ± 1.67	1.97 ± 1.23	1.82 ± 0.27	1.27 ± 0.9	2.21 ± 0.56

Table S3. Reference values of bulk skeletal density and calculated porosity for the seven scleractinian coral genera. Values are grouped by host evolutionary lineage. Density data were retrieved from published literature, prioritizing studies conducted in the Luhuitou Reef to minimize geographic variation.

Host Clade	Coral Genus	Skeletal Density (g/cm^3, Mean ± SD)	Porosity (%, Calculated)	Reference Source
Complex	*Acropora* sp.	1.57 ± 0.34	46.4 ± 11.6	[1]
Complex	*Astreopora* sp.	1.48	49.5	[1]
Complex	*Goniopora* sp.	1.31 ± 0.16	55.3 ± 5.5	[1]
Complex	*Porites* sp.	1.31 ± 0.16	55.3 ± 5.5	[1]
Robust	*Dipsastraea* sp.	1.26 ± 0.07	57.0 ± 0.002	[1]
Robust	*Favites* sp.	1.24 ± 0.15	57.7 ± 5.1	[1]
Robust	*Platygyra* sp.	1.22 ± 0.17	58.4 ± 5.8	[1]

Note: Porosity was calculated based on the bulk skeletal density using the formula: Porosity (%) = (1−Density/2.93)×100, assuming the density of pure aragonite is 2.93 g/cm^3[2,3]. Where standard deviation (SD) was not reported in the source, only the mean is provided.

Table S4. Statistical tests linking skeletal porosity with microbial community structure (Beta-diversity). Mantel tests correlate Bray-Curtis dissimilarity matrices with Euclidean distance matrices of skeletal porosity. PERMANOVA (Adonis) tests the marginal effects of host clade and porosity on community structure.

Domain	Test Method	Formula / Variables	Statistic	Value	P-value
Bacteria	Mantel Test	Community ~ Porosity	Spearman's r	0.188	0.027
	PERMANOVA	Dist ~ Clade + Porosity	R^2	0.130	0.001
Archaea	Mantel Test	Community ~ Porosity	Spearman's r	0.078	0.140
	PERMANOVA	dist ~ Clade + Porosity	R^2	0.145	0.018

Table S5. Mean relative abundances (±%) of dominant bacterial phyla across different coral genera and evolutionary clades.

Phylum	Complex (Mean)	Robust (Mean)	*Acropora* sp.	*Astreopora* sp.	*Goniopora* sp.	*Porites* sp.	*Dipsastraea* sp.	*Favites* sp.	*Platygyra* sp.
Pseudomonadota	34.8 ± 15.1%	45.7 ± 19.0%	37.3 ± 32.7%	37.5 ± 6.3%	27.7 ± 10.8%	35.7 ± 4.0%	36.6 ± 19.2%	67.3 ± 9.9%	35.6 ± 7.4%
Planctomycetota	13.7 ± 9.3%	13.9 ± 7.6%	19.6 ± 19.5%	9.7 ± 0.7%	14.4 ± 7.0%	12.5 ± 1.6%	20.6 ± 9.3%	6.5 ± 1.8%	14.5 ± 2.5%
Bacillota	14.4 ± 14.3%	8.9 ± 3.7%	12.3 ± 5.3%	8.4 ± 1.1%	26.2 ± 29.7%	12.7 ± 3.2%	8.8 ± 1.8%	5.8 ± 4.2%	11.5 ± 2.6%
Bacteroidota	7.1 ± 3.0%	6.9 ± 5.6%	5.7 ± 3.3%	9.7 ± 3.4%	6.5 ± 1.8%	5.8 ± 0.9%	4.8 ± 2.8%	9.0 ± 9.8%	6.9 ± 2.6%
Actinomycetota	2.5 ± 1.4%	7.0 ± 9.5%	1.6 ± 1.9%	3.0 ± 1.5%	1.7 ± 0.7%	3.5 ± 0.6%	15.0 ± 14.9%	1.8 ± 1.6%	4.7 ± 1.2%
Chloroflexota	5.2 ± 3.0%	2.7 ± 2.2%	5.2 ± 4.2%	5.1 ± 3.3%	4.0 ± 3.5%	6.5 ± 1.4%	2.6 ± 2.4%	1.6 ± 2.7%	3.7 ± 1.6%
Verrucomicrobiota	4.0 ± 2.7%	3.3 ± 2.4%	1.3 ± 1.9%	5.3 ± 4.0%	4.5 ± 0.7%	4.7 ± 0.9%	2.5 ± 1.7%	1.1 ± 0.8%	5.6 ± 1.5%
Babelota	4.4 ± 6.3%	1.3 ± 1.2%	4.9 ± 4.0%	8.2 ± 10.6%	1.0 ± 0.6%	2.5 ± 1.6%	0.8 ± 1.2%	1.0 ± 1.5%	1.9 ± 1.0%
Acidobacteriota	2.9 ± 1.8%	1.7 ± 1.6%	2.4 ± 2.1%	1.8 ± 0.8%	2.9 ± 2.4%	4.8 ± 0.6%	2.0 ± 2.4%	0.4 ± 0.2%	2.4 ± 0.9%
Patescibacteriota	2.4 ± 1.4%	1.4 ± 1.6%	1.8 ± 1.2%	2.6 ± 0.7%	3.1 ± 2.7%	2.2 ± 1.1%	0.3 ± 0.5%	2.9 ± 2.4%	1.2 ± 0.5%
Desulfobacterota	1.2 ± 0.9%	1.1 ± 1.2%	0.8 ± 0.6%	1.6 ± 1.3%	1.3 ± 1.0%	1.1 ± 0.0%	1.8 ± 1.6%	0.0 ± 0.1%	1.3 ± 0.7%
Others	7.3 ± 2.8%	6.2 ± 4.9%	7.1 ± 5.1%	7.2 ± 3.4%	6.8 ± 1.3%	8.0 ± 1.2%	4.1 ± 1.4%	2.8 ± 3.5%	10.7 ± 4.6%

Table S6. Mean relative abundances (±%) of dominant archaeal phyla across different coral genera and evolutionary clades.

Phylum	Complex (Mean)	Robust (Mean)	*Acropora* sp.	*Astreopora* sp.	*Goniopora* sp.	*Porites* sp.	*Dipsastraea* sp.	*Favites* sp.	*Platygyra* sp.
Nanoarchaeota	58.2 ± 28.3%	73.7 ± 21.6%	53.9 ± 18.6%	69.1 ± 37.1%	44.2 ± 27.6%	61.9 ± 32.4%	51.1 ± 25.4%	85.9 ± 11.0%	82.2 ± 9.1%
Thermoproteota	39.8 ± 27.7%	24.4 ± 21.2%	44.4 ± 19.5%	30.3 ± 37.2%	52.5 ± 27.0%	35.1 ± 30.4%	46.8 ± 24.9%	12.9 ± 9.3%	15.6 ± 9.0%
Halobacterota	0.5 ± 1.2%	1.2 ± 2.0%	0.4 ± 0.4%	0.1 ± 0.1%	1.5 ± 2.5%	0.1 ± 0.2%	1.7 ± 2.9%	1.1 ± 2.1%	1.0 ± 1.3%
Aenigmarchaeota	0.5 ± 1.0%	0.1 ± 0.3%	1.2 ± 1.3%	0.0 ± 0.0%	0.0 ± 0.0%	1.1 ± 1.4%	0.3 ± 0.5%	0.0 ± 0.0%	0.0 ± 0.0%
Euryarchaeota	0.2 ± 0.8%	0.1 ± 0.2%	0.0 ± 0.0%	0.0 ± 0.0%	1.0 ± 1.7%	0.0 ± 0.0%	0.0 ± 0.0%	0.0 ± 0.1%	0.3 ± 0.3%
Thermoplasmatota	0.2 ± 0.5%	0.1 ± 0.3%	0.0 ± 0.0%	0.1 ± 0.1%	0.8 ± 0.9%	0.1 ± 0.2%	0.0 ± 0.0%	0.0 ± 0.1%	0.2 ± 0.5%
Iainarchaeota	0.1 ± 0.4%	0.0 ± 0.0%	0.0 ± 0.0%	0.0 ± 0.0%	0.0 ± 0.0%	0.5 ± 0.9%	0.0 ± 0.0%	0.0 ± 0.0%	0.0 ± 0.0%
Asgardarchaeota	0.0 ± 0.1%	0.1 ± 0.2%	0.0 ± 0.0%	0.0 ± 0.0%	0.1 ± 0.1%	0.0 ± 0.0%	0.0 ± 0.0%	0.0 ± 0.0%	0.1 ± 0.3%
Altiarchaeota	0.0 ± 0.1%	0.0 ± 0.0%	0.0 ± 0.0%	0.0 ± 0.0%	0.1 ± 0.1%	0.0 ± 0.0%	0.0 ± 0.0%	0.0 ± 0.0%	0.0 ± 0.0%

Table S7. Topological properties of cross-domain co-occurrence networks constructed from endolithic bacterial and archaeal communities in complex and robust coral clades.

	Complex	Robust
Nodes	248	230
Edges	2649	3127
Average degree	21.363	27.191
Graph density	0.086	0.119
Modularity	0.428	0.474
Positive links	99.92%	99.97%
Average Clustering Coefficient	0.523	0.638
Average path length	3.111	3.329

Table S8. Topological roles and connectivity scores (Z_i and P_i) of microbial nodes identified in the co-occurrence networks of complex and robust coral clades.

Group	Domain	Phylum	Role	Zi	Pi
Complex	Bacteria	Chloroflexota	Connectors	-1.27	0.63
Complex	Bacteria	Bacillota	Connectors	-1.41	0.66
Complex	Bacteria	Pseudomonadota	Connectors	-1.47	0.63
Complex	Bacteria	Acidobacteriota	Connectors	-1.68	0.62
Robust	Bacteria	Bacteroidota	Connectors	-1.59	0.65

Table S9. Detailed information of the functional marker genes used for metabolic potential prediction in PICRUSt2 analysis.

Metabolic Pathway	Functional Gene	KO Number	Name
Nitrogen fixation	*NifH*	K02588	nitrogenase iron protein NifH
	NifD	K02586	nitrogenase molybdenum-iron protein alpha chain [EC:1.18.6.1]
	NifK	K02591	nitrogenase molybdenum-iron protein beta chain [EC:1.18.6.1]
	anfG	K00531	nitrogenase delta subunit [EC:1.18.6.1]
Nitrification	*amoB*	K22359	alkene monooxygenase gamma subunit [EC:1.14.13.69]
	amoC	K22360	alkene monooxygenase ferredoxin subunit
	hao	K10535	hydroxylamine dehydrogenase [EC:1.7.2.6]
Denitrification	*narG*	K00370	nitrate reductase / nitrite oxidoreductase, alpha subunit [EC:1.7.5.1 1.7.99.-]
	narH	K00371	nitrate reductase / nitrite oxidoreductase, beta subunit [EC:1.7.5.1 1.7.99.-]
	narI	K00374	nitrate reductase gamma subunit [EC:1.7.5.1 1.7.99.-]
	napA	K02567	nitrate reductase (cytochrome) [EC:1.9.6.1]
	napB	K02568	nitrate reductase (cytochrome), electron transfer subunit
	nirK	K00368	nitrite reductase (NO-forming) [EC:1.7.2.1]
	nirS	K15864	nitrite reductase (NO-forming) / hydroxylamine reductase [EC:1.7.2.1 1.7.99.1]
	norB	K04561	nitric oxide reductase subunit B [EC:1.7.2.5]
	norC	K02305	nitric oxide reductase subunit C
	nosZ	K00376	nitrous-oxide reductase [EC:1.7.2.4]
Sulfur metabolism	*dsrA*	K11180	dissimilatory sulfite reductase alpha subunit [EC:1.8.1.22]
	soxB	K17224	S-sulfosulfanyl-L-cysteine sulfohydrolase [EC:3.1.6.20]

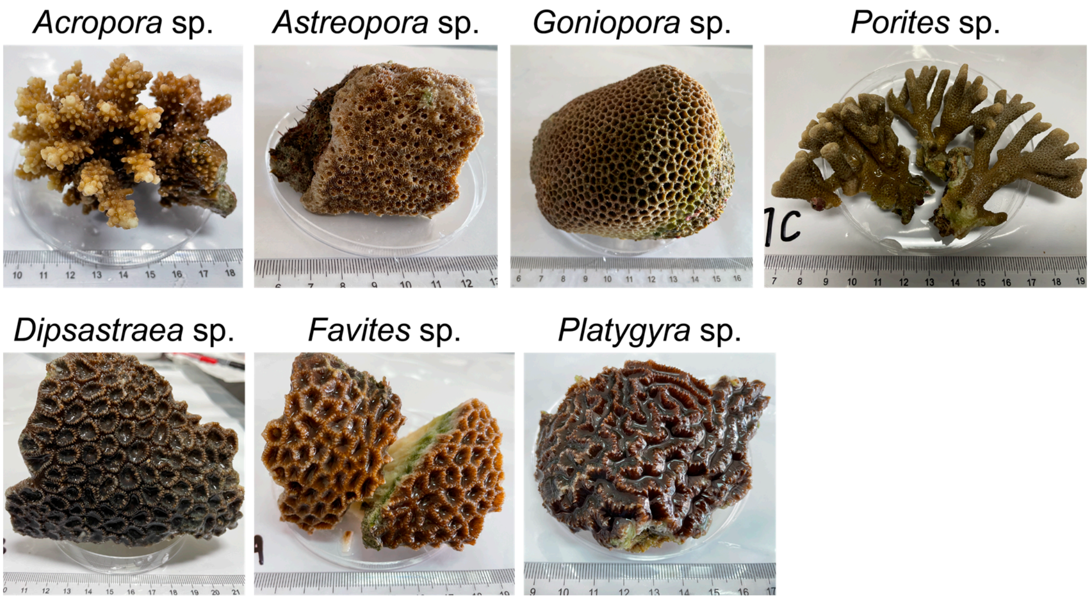

Figure S1. Morphological features of the seven sampled coral genera. *Acropora* sp.: Fast-growing, branching morphology with a high surface-to-volume ratio; typically found in high-energy, well-lit reef zones (e.g., reef crests). *Astreopora* sp.: Massive to encrusting forms characterized by star-shaped corallites; generally occurs in diverse reef environments, ranging from shallow lagoons to clear reef slopes. *Goniopora* sp.: Sub-massive, columnar, or encrusting forms; often found in turbid waters or lagoonal environments with moderate water flow. *Porites* sp.: Predominantly massive and slow-growing; highly resilient and long-lived, capable of thriving in a wide range of environments from inner reef flats to deep slopes. *Dipsastraea* sp.: Massive or sub-massive morphology with distinct, large corallites; broadly distributed across various reef zones and adapted to diverse light conditions. *Favites* sp.: Robust massive or encrusting forms with shared corallite walls (cerioid); known for high resistance to sedimentation and environmental stress. *Platygyra* sp.: Massive "brain coral" with meandroid (labyrinth-like) ridges; well-adapted to inner reef flats and environments with high sedimentation or variable salinity. Morphological and ecological descriptions are based on Veron (2000) and the Corals of the World database [4].

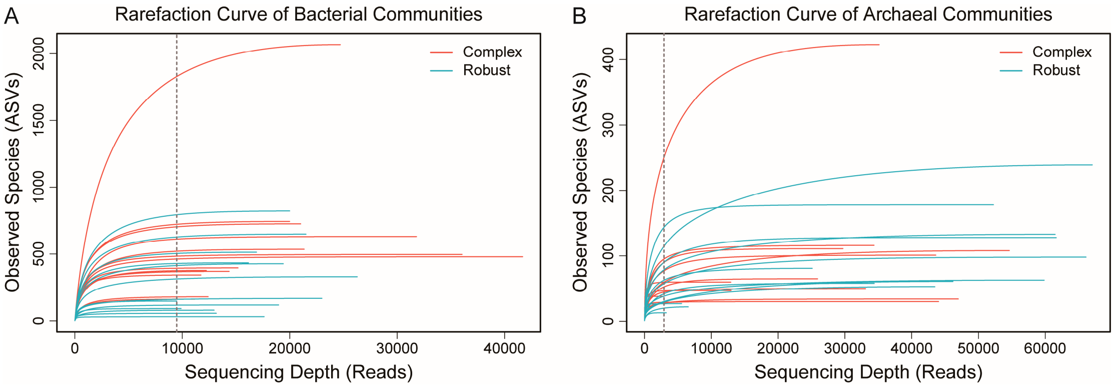

Figure S2. Rarefaction curves of 16S rRNA gene sequences for endolithic bacterial and archaeal communities. Plots show the number of observed ASVs as a function of sequencing depth (number of reads) for (A) bacterial and (B) archaeal samples. Curves for both Complex (red lines) and Robust (blue lines) clades reach a saturation plateau, indicating that the sequencing depth was sufficient to capture the majority of microbial diversity in the coral skeletal samples. Vertical dashed lines indicate the rarefaction depth used for downstream diversity analyses (Bacteria: 9,480 reads; Archaea: 2,913 reads).

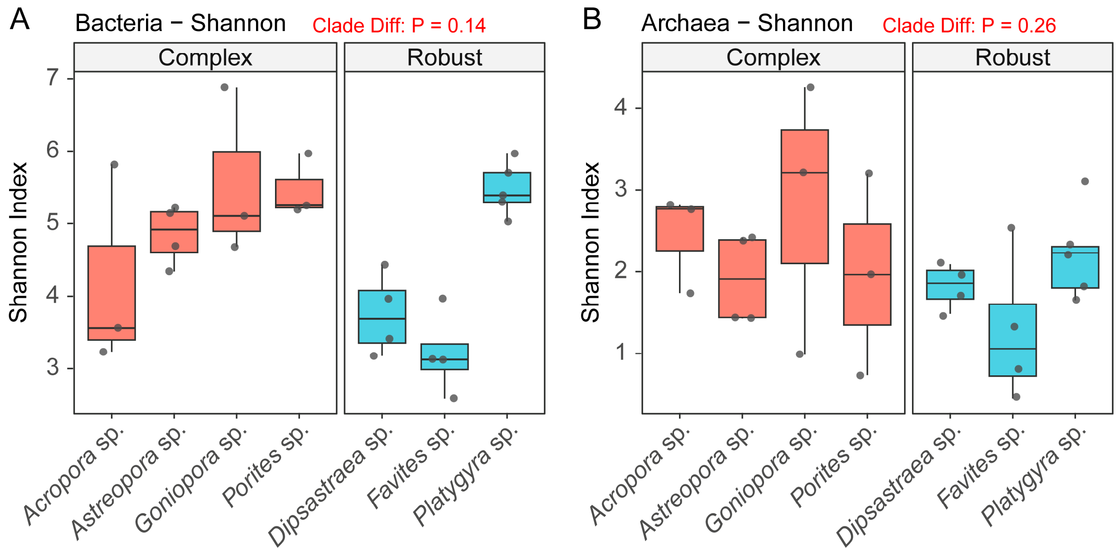

Figure S3. Shannon diversity indices of endolithic microbial communities compared between host evolutionary lineages. Boxplots represent the Shannon diversity index (accounting for both richness and evenness) for (A) bacteria and (B) archaea. Samples are grouped by host clade, Complex (red) and Robust (blue). Statistical significance was assessed using the Wilcoxon rank-sum test. Consistent with the Chao1 richness results (Figure 2), no statistically significant difference was observed for Shannon diversity in either bacteria ($P = 0.14$) or archaea ($P = 0.26$).

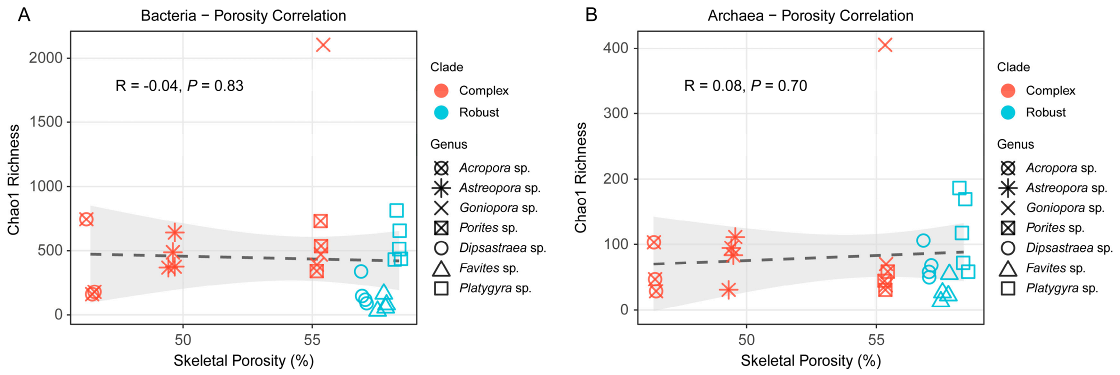

Figure S4. Correlation analysis between coral skeletal porosity and microbial alpha diversity. Scatter plots showing the relationship between calculated skeletal porosity (%) and Chao1 richness for (A) bacterial and (B) archaeal communities. Each point represents an individual coral sample, with shapes indicating coral genera and colors indicating evolutionary clades. Skeletal porosity was derived from density values obtained from literature regarding corals in the Luhuitou Reef area (see Table S7). Pearson correlation coefficients (R) and P-values are displayed. Shaded areas represent 95% confidence intervals. The lack of strong statistical significance ($P > 0.05$) indicates that while porosity may influence bacterial colonization potential, it is not the sole determinant, with host-specific factors likely playing a stronger role.

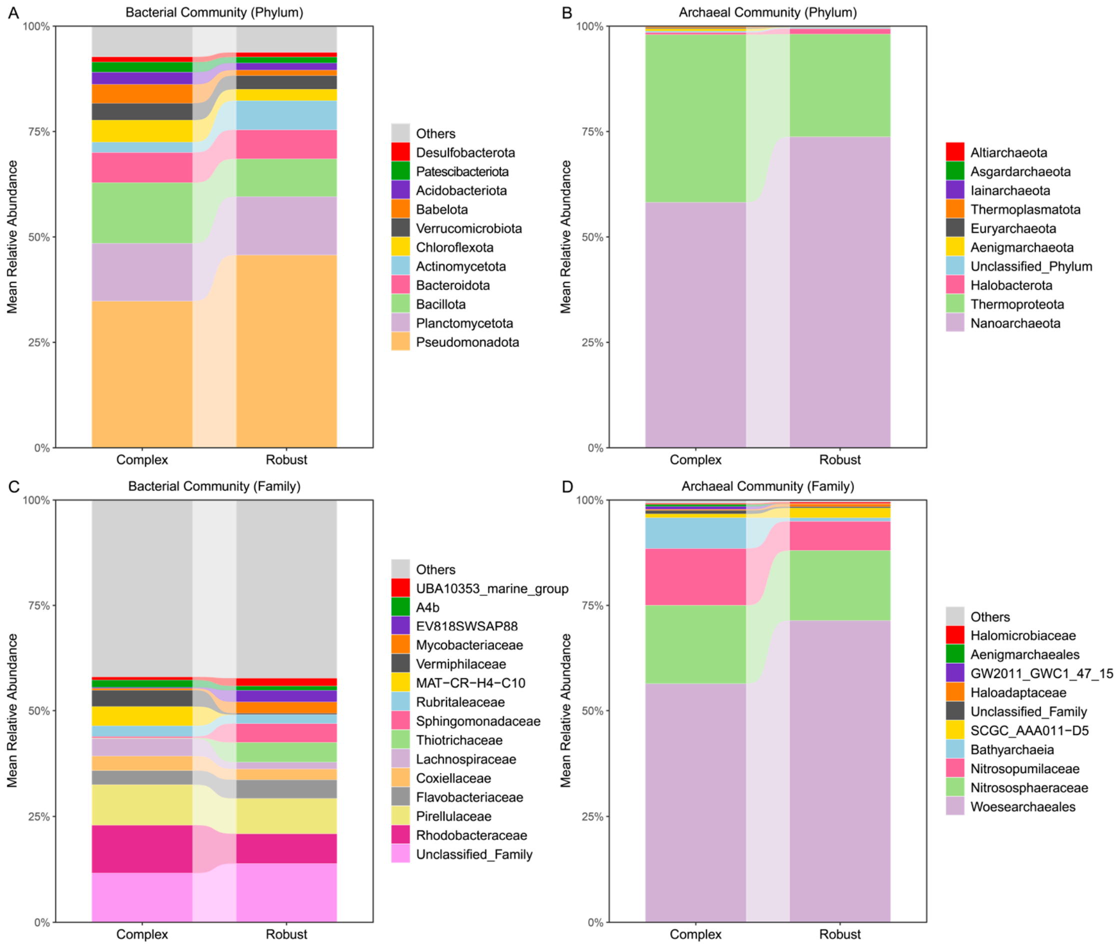

Figure S5. Taxonomic composition of endolithic communities at both phylum and family levels. Alluvial plots illustrate the mean relative abundance of dominant (A) bacterial and (B) archaeal phyla, as well as (C) bacterial and (D) archaeal families, aggregated by host evolutionary lineage (Complex vs. Robust). The flows connect the same taxa between the two clades.

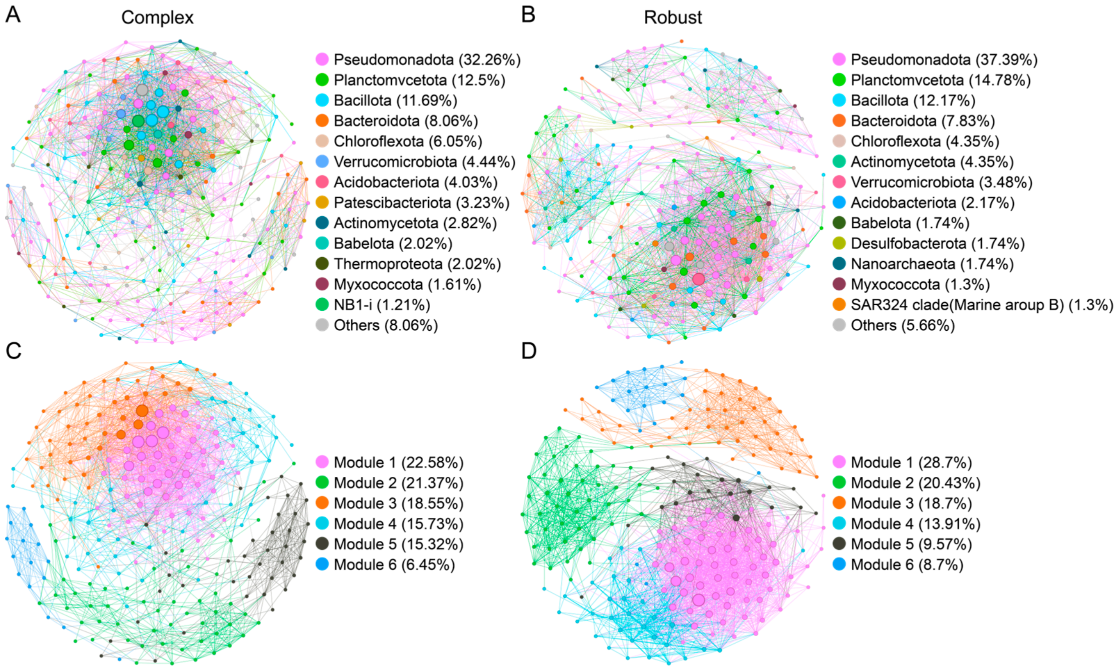

Figure S6. Visualization of cross-domain co-occurrence networks colored by taxonomic affiliation and modular structure. The networks were constructed based on significant Spearman correlations (|r| > 0.6, $P < 0.05$) of endolithic bacterial and archaeal ASVs. (A, B) Network graphs for Complex (A) and Robust (B) clades, with nodes colored according to their Phylum assignment. The legend displays the percentage of nodes belonging to each phylum within the network. Note the high prevalence of Pseudomonadota and Planctomycetota in both networks. (C, D) The same networks for Complexa (C) and Robusta (D) clades, but with nodes colored based on modularity classes (densely connected sub-communities) identified by the fast-greedy modularity algorithm. The legend indicates the percentage of nodes assigned to each module. In all panels, the size of each node is proportional to its degree (number of connections).

References

1. Shi, Q.; Zhao, M.; Zhang, Q.; Yu, K.; Chen, T.; Li, S.; Wang, H. Estimate of Carbonate Production by Scleractinian Corals at Luhuitou Fringing Reef, Sanya, China. *Chin. Sci. Bull.* **2009**, *54*, 696–705, doi:10.1007/s11434-008-0533-9.
2. Bucher, D.J.; Harriott, V.J.; Roberts, L.G. Skeletal Micro-Density, Porosity and Bulk Density of Acroporid Corals. *Journal of Experimental Marine Biology and Ecology* **1998**, *228*, 117–136, doi:10.1016/S0022-0981(98)00020-3.
3. Caroselli, E.; Prada, F.; Pasquini, L.; Marzano, F.N.; Zaccanti, F.; Falini, G.; Levy, O.; Dubinsky, Z.; Goffredo, S. Environmental Implications of Skeletal Micro-Density and Porosity Variation in Two Scleractinian Corals. *Zoology* **2011**, *114*, 255–264, doi:10.1016/j.zool.2011.04.003.
4. Veron, J.E.N. *Corals of the World*; Australian Institute of Marine Science, 2000; ISBN 978-0-642-32236-4.
